# Predicting rock–paper–scissors choices based on single‐trial EEG signals

**DOI:** 10.1002/pchj.688

**Published:** 2023-10-31

**Authors:** Zetong He, Lidan Cui, Shunmin Zhang, Guibing He

**Affiliations:** ^1^ Department of Psychology and Behavioral Sciences Zhejiang University Hangzhou China; ^2^ College of Computer Science and Technology, Zhejiang University Hangzhou China

**Keywords:** attractor metagene, common spatial pattern, decision making, electroencephalogram (EEG), single‐trial prediction

## Abstract

Decision prediction based on neurophysiological signals is of great application value in many real‐life situations, especially in human–AI collaboration or counteraction. Single‐trial analysis of electroencephalogram (EEG) signals is a very valuable step in the development of an online decision‐prediction system. However, previous EEG‐based decision‐prediction methods focused mainly on averaged EEG signals of all decision‐making trials to predict an individual's general decision tendency (e.g., risk seeking or aversion) over a period rather than on a specific decision response in a single trial. In the present study, we used a rock–paper–scissors game, which is a common multichoice decision‐making task, to explore how to predict participants' single‐trial choice with EEG signals. Forty participants, comprising 20 females and 20 males, played the game with a computer player for 330 trials. Considering that the decision‐making process of this game involves multiple brain regions and neural networks, we proposed a new algorithm named common spatial pattern‐attractor metagene (CSP‐AM) to extract CSP features from different frequency bands of EEG signals that occurred during decision making. The results showed that a multilayer perceptron classifier achieved an accuracy significantly exceeding the chance level among 88.57% (31 of 35) of participants, verifying the classification ability of CSP features in multichoice decision‐making prediction. We believe that the CSP‐AM algorithm could be used in the development of proactive AI systems.

## INTRODUCTION

Predicting decision‐making choices and tendencies is of great application value in many fields. This ability helps robots recognize human intention and offer active and adaptive assistance in human–robot collaboration (HRC; Awais & Henrich, 2010). In a game scenario, it helps a player anticipate their opponent's intentions and formulate a specific strategy (L. Wang et al., 2020). Online decision prediction systems have long been the goal of human–computer interaction (HCI) systems, as they have the potential to help in the development of adaptive human–computer interfaces that can react to a user's mental state (Lotte et al., 2018; Zander & Kothe, 2011). In addition to behavioral data, which are used to predict decision responses by referring to a decision‐maker's strategy, neurophysiological signals, such as electroencephalogram (EEG) and functional magnetic resonance imaging (fMRI), contain important information for decision prediction (Si et al., 2019). Among the different kinds of neurophysiological signals, EEG plays an irreplaceable role in real‐time decision prediction owing to its high temporal resolution and easy setup. Notably, there have been EEG‐based brain–computer interfaces (BCIs) for the online prediction of motor imagery (Tabar & Halici, 2017; Xu & Li, 2022), speech synthesis (Anumanchipalli et al., 2019), emotion recognition (Z. Wang et al., 2022), and many other applications (Wolpaw et al., 2002). However, in relation to more complicated decision‐making scenarios, such an EEG‐based online prediction system has not yet been well developed.

Building an online decision‐prediction framework helps us better understand the decision‐making process (Bhushan et al., 2012; Page, 2012) and has application value in many aspects of daily life (Chong et al., 2012; Sun et al., 2018; Suri et al., 2013). Single‐trial analysis is necessary in online decision prediction owing to its potential in establishing biologically inspired AI systems (Hsu, 2013; Si et al., 2019). However, previous decision‐prediction research has focused mainly on averaged EEG signals rather than on single‐trial signals. These averaged measurements could be used to predict overall individual tendencies, such as impulsivity (Lv et al., 2019) and short‐sighted preference (Chen et al., 2019), but trial‐to‐trial information would be lost, and the varied states of individuals would not be reflected (Ratcliff et al., 2009). Consequently, researchers have been unable to conduct single‐trial decision‐making predictions based on averaged measurements of EEG signals or to develop online decision‐prediction systems. Although a few studies have focused on predicting individual decision‐making responses based on single‐trial EEG signals (Si et al., 2020), the paradigms they adopted, such as the ultimatum game (Güth et al., 1982), are uncommon in real‐life situations. Thus, these studies contribute little to applications of real‐time BCIs in real‐life situations.

In this study, we adopted the rock–paper–scissors game as an experimental task because of its three main advantages. First, the rock–paper–scissors game is a multichoice decision task, which is more common than the binary decision task included in HRC and multiparty games. Previous studies have shown that decision making with multiple choices has a different mechanism than binary decision making (Busemeyer et al., 2019; Ditterich, 2010). Approaches predicting multichoice decision‐making responses can be applied in more real‐life situations, but few such studies of this nature have been done because it is algorithmically harder to solve multiclass classification problems than binary problems (Lorena et al., 2008). To the best of our knowledge, few studies have focused on multichoice decision prediction based on single‐trial EEG signals. Second, the rock–paper–scissors game is popular in daily life among children and adults for making decisions on trivial disputes that have no obvious optimal choice (Szolnoki et al., 2014). Third, it is a decision‐making task that does not result in feelings such as anger and impulsivity or a sense of injustice and rejection, which are feelings that are elicited in the ultimatum game. Prediction models obtained in such paradigms should be a good supplement to other decision‐making scenarios in daily life that do not involve many emotions.

The spatial features of EEG signals might be of vital significance in single‐trial decision‐making predictions. Previous studies have demonstrated that decision making involves many brain regions and neural networks. For example, it was found that the ventromedial prefrontal cortex (vmPFC), anterior cingulate cortex (ACC), insula, and dorsolateral prefrontal cortex (DLPFC) play important roles in the decision‐making process (Bartra et al., 2013; Critchley et al., 2000; Krain et al., 2006; Suzuki et al., 2012; Tanabe et al., 2007). Furthermore, the interactions among these brain regions contribute to the decision‐making process. For example, the association between the right insula and the inferior frontal cortex contributes to the integration of sensory information with response‐related information in goal‐directed behavior (Dodds et al., 2011). In addition to the spatial features of EEG signals, different frequency bands of EEG signals have been found to carry different aspects of information during the decision‐making process. For example, when a person is making complex decisions, the EEG spectrum significantly decreases in the alpha band, but the spectrum significantly increases in the delta, theta, beta and gamma bands (Davis et al., 2011). Therefore, we developed a multivariate trial‐by‐trial classification framework that extracted spatial features in different frequency bands of neural signals during the decision‐making process.

In sum, we aimed to adopt an EEG‐based machine learning (ML) algorithm to predict individual trial‐by‐trial responses in a rock–paper–scissors game. EEG datasets were collected when participants were playing a rock–paper–scissors game with a computer player. Thereafter, we divided the EEG data into five frequency bands of 1–4, 4–8, 8–12, 12–16 and 16–20 Hz to extract more frequency‐specific information. Common spatial pattern (CSP) features were then extracted in every frequency band and concatenated to form a high‐dimensional feature set. The attractor metagene (AM) algorithm was utilized to reduce the dimension, and a one‐versus‐one (OVO) strategy was applied to extend the CSP from binary problems to ternary problems. Some common classifiers with relatively high time efficiency were trained for the single‐trial prediction of individual responses in the rock–paper–scissors game.

The remainder of this paper is organized as follows. The second part introduces the data acquisition process and the method utilized for feature extraction, selection, and classification. The third part reports the results of our experiment. The fourth and fifth parts discuss the results and summarize the paper, respectively.

## MATERIALS AND METHODS

### Machine learning in EEG studies

ML involves the use of a set of mathematical models and algorithms to gradually improve the performance of a singular task (Hosseini et al., 2021). ML can be categorized into two main types: supervised learning and unsupervised learning. In supervised learning, a model is trained with labeled data and learns to predict an output based on the input features. In unsupervised learning, however, a model is trained with unlabeled data and learns to identify patterns and structures in the data without any specific output to predict (Sathya & Abraham, 2013). In supervised learning, the dataset is usually divided into a training set and a testing set. The training set is used to train the model, while the testing set is used to evaluate the model's performance on unseen data.

**FIGURE 1:**
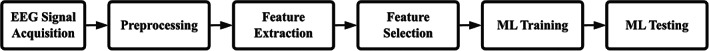
General process of machine learning (ML) in electroencephalogram (EEG) studies.

Figure 1 describes the general process of how ML is implemented to obtain the desired classification of EEG signals. During signal acquisition, the raw EEG data are collected, while during preprocessing, noise and other outliers in the dataset are removed. Feature extraction is the process of obtaining relevant information from EEG signals to represent the underlying physiological or cognitive processes. Feature selection is the process of selecting the subset of features that are most relevant to the classification task. ML training uses the training set to refine the classification model, while ML testing evaluates the model's performance on the testing set.

### Participants

Forty participants (20 females, age range: 18–27 years, mean age: 22.1 years) were recruited for this study. None of them had a history of neurological or psychiatric disorders, and they were all native Chinese speakers with normal or corrected‐to‐normal vision. The experimental protocol was approved by the Research Ethics Committee of Zhejiang University, and informed consent was obtained from all participants before they engaged in this study.

### Experimental protocol

We adopted the rock–paper–scissors game to simulate a more ecological and multichoice decision‐making scenario. The experiments were performed in a quiet room. In the first few minutes, participants were instructed to take a deep breath and to adapt to the experimental environment. Then, the participant started with a practice session of 30 trials to become familiar with the task and the keys to be pressed. Before the formal session began, we informed the participants that they needed to play a rock–paper–scissors game against a computer player whose decision followed a certain rule (though the decision was in fact random). We asked the participants to win as many games as they could, and if they won more than the computer, a ¥10 bonus would be added to their base payment.

The formal session contained 330 trials (10 blocks, each containing 33 trials) and lasted ~1.5 h. During each block, the participants were instructed to reduce their body movements and just move their fingers to make the response. The participants took a break between blocks until they were ready for the next block. After participants finished all the blocks, a brief interview was conducted to investigate participants' actual thought processes during the experiment.

**FIGURE 2:**
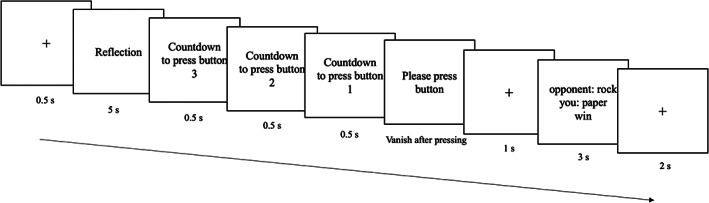
Timeline of the rock–paper–scissors game.

Figure 2 shows the timeline of the rock–paper–scissors game. At the start of each trial, a fixation cross appeared for 0.5 s. The reflection stage lasted for 5 s and allowed participants to consider their choice and make their decision. Then, a countdown from three to one appeared to remind the participant to become ready to press a button (“J”, “K” and “L” keys on the keyboard, each representing a choice of either rock, paper or scissors, balanced among participants). Then, the phrase “Please press a button” was shown on the screen and vanished as soon as the participant pressed a button. After this, a fixation cross was presented for 1 s, and feedback was shown on the screen to inform the participant of the result of this turn. A fixation cross was then shown for 2 s, indicating that the next trial was imminent.

### EEG data acquisition and preprocessing

Continuous EEG signals were recorded from 34 scalp sites using active Ag–AgCl electrodes embedded in an elastic cap, at locations according to the modified expanded 10–20 system (Biosemi ActiveTwo, Amsterdam, the Netherlands). EEG data were sampled at 512 Hz. The electrooculogram (EOG) was recorded using four additional facial electrodes: two electrodes placed ~1 cm outside the right and left eyes, and two electrodes mounted ~1 cm above and below the right eye. A common mode sense (CMS) active electrode producing a monopolar (i.e., nondifferential) channel was used as a recording reference.

Trials with reaction times (i.e., display time of “Please press a button”) outside three standard deviations from the mean (approximately less than 100 ms or greater than 1500 ms) were removed from data analysis. After this step, the data of five participants were removed from the analysis because the number of their remaining trials was less than 1/3 of the total number of trials. The EEG signals were preprocessed in MATLAB 2019a using the EEGLAB toolbox (Delorme & Makeig, 2004). The data were referenced to the average of the left and right mastoids and bandpass filtered with low and high cutoffs of 1 and 20 Hz, respectively. Epochs containing artifacts outside the −75 to 75 μV range were rejected. Signals within a time window of 1500 ms before the participant pressed the button were extracted. To detect and correct blinking and motion artifacts, we then used independent component analysis (ICA), which is effective in detecting and removing eye‐related contamination (Delorme & Makeig, 2004). Finally, each subject's EEG data were divided into frequency bands of 1–4, 4–8, 8–12, 12–16, and 16–20 Hz. Previous studies demonstrated that good performance can be obtained using these frequency ranges in decision‐prediction tasks (Si et al., 2020), and such 4‐Hz‐interval frequency bands yielded a stable frequency response (Ang et al., 2012; Park & Chung, 2019; Selim et al., 2018) in EEG classification problems that used CSP features.

### Feature extraction

As mentioned in Section 1, the spatial features of EEG signals may be of vital significance in single‐trial decision‐making predictions. As a feature extraction method in the spatial domain, CSP has the potential to distinguish different spatial patterns for different decision‐making responses. Moreover, CSPs have been shown to be extremely useful in single‐trial analysis to improve the signal‐to‐noise ratio and have been shown to be a powerful technique in BCI research for distinguishing different mental intentions (Blankertz et al., 2008).

A CSP decomposes two populations of multivariate signals into a set of spatial patterns, in which the differences in variances between the signals of the two populations are maximized (MuÈller‐Gerking et al., 1999). However, a CSP can only address binary classification problems. Hence, in this study, the multichoice problem had to be divided into binary problems using a classification strategy. The basic CSP algorithm was devised as follows.

Spatial filters are applied to a binary problem, where they maximize the variance in one class while minimizing the variance in the other. The two classes are denoted by $c=\left(x,y\right)$. Let $X^{1},\ldots ,X^{m}\in R^{n\times l}$ be the trials of class x, and let $Y^{1},\ldots ,Y^{m}\in R^{n\times l}$ be the trials of class y, where n represents the number of channels and l represents the number of sample points per trial. Each class has M trials for training, denoted by m, where m=1,2,…,M (Blankertz et al., 2008).

Let matrix E of size n×l represent a single trial. The sample covariance matrix S of a given trial E is normalized with the total variance, as shown below:

(1) $$S=\frac{EE^{T}}{\mathrm{tr}\left({\mathrm{EE}}^{T}\right)},$$

where the superscript “T” denotes the transposed matrix, and $\mathrm{tr}\left(∙\right)$ is the trace of the matrix.

Hence, $E\left(c,m\right)$ can be denoted as the matrix of given class c and trial m. Each trial has its covariance matrix $S\left(c,m\right)$. Thus, the average spatial covariance matrix can be calculated for each class as follows:

(2) $$\begin{array}{c} {\bar{S}}_{c}=\frac{1}{M}\sum_{m=1}^{M}S_{\left(c,m\right)}. \end{array}$$

The discriminative spatial patterns in CSP are extracted from the estimation of the above sample covariance matrix (Lu et al., 2010). High variance indicates one class, while low variance indicates the other.

In this study, we extracted CSP features for all five frequency bands. For each band, four pairs of spatial filters were selected, because this number of filter pairs strikes a balance between the classification capability and the number of dimensions (Ang et al., 2008; Pei et al., 2022; Selim et al., 2018). The resulting CSP selected features for all five bands were concatenated, forming 40‐dimensional (5 bands× 4 spatial pairs) features.

### Feature selection

AM algorithms were applied for feature selection in this study. The AM is an unsupervised algorithm, and its efficiency has been shown in prognostic models of breast cancer (Ou Yang et al., 2014). One of its major advantages is that it is based on mutual information estimation to calculate statistical dependence among given variables. As a hybrid feature‐selection model, the AM technique has been shown to be greatly resistant to overfitting compared with filter models (Liu & Yu, 2005). The AM algorithm is described briefly as follows.

The weights of each feature are randomly initialized. The estimated metagene is recalculated in each iteration. The $i^{\mathrm{th}}$ iteration is calculated as follows:

(3) $$\begin{array}{c} M_{i}=w_{i}\times G, \end{array}$$

$\text{where}\;w_{i}$ is a vector of weights of size 1×p, where p is the number of genes, and G is the gene expression matrix of size p×n, where *n* denotes the number of samples. The weights are updated every $i^{\mathrm{th}}$ iteration according to the measure of similarity by

(4) $$\begin{array}{c} W_{j,i+1}=J\left(M_{i},G_{j}\right), \end{array}$$

where $W_{j,i+1}$ is the $j^{\mathrm{th}}$ element of $w_{i+1}$ and $G_{j}$ is the $j^{\mathrm{th}}$ row of G. The similarity metric j is defined by calculating the Pearson correlation between $M_{i}$ and $G_{j}$; if the Pearson correlation is greater than 0, then

(5) $$\begin{array}{c} J\left(M_{i},G_{j}\right)=I\left(M_{i},G_{j}\right)\alpha , \end{array}$$

where $I\left(M_{i},G_{j}\right)$ is the amount of mutual information between two genes, ranging between 0 and 1, and α is defined as any nonnegative number.

Moreover, if the Pearson correlation is less than or equal to 0, then

(6) $$\begin{array}{c} J\left(M_{i},G_{j}\right)=0, \end{array}$$

and the algorithm iterates until the change in $w_{i}$ along the iterations becomes less than the predefined tolerance or the maximum number of iterations is reached.

In this study, the AM algorithm was used to derive a weight for each CSP feature. The features were then sorted in descending order according to their AM weights. The training process was iterated 40 times. In the $k^{\mathrm{th}}$ iteration, the first k‐sorted features were introduced to the classifier. That is, the features were added to the classifier one by one. The aim was to use as few features as possible in order to achieve better performance. The subset with minimum k‐features that achieved the best accuracy was selected to be the best feature subset for the given participant's pair of classes.

### One‐versus‐one (OVO) classification strategy

The OVO decomposition strategy (Knerr et al., 1990) is one of the most commonly used strategies to expand binary classification problems to multiclass problems (Galar et al., 2011). A previous study also used the OVO strategy to apply CSP in multiclass motor imagery tasks (Selim et al., 2018). As noted above, CSP can only address the binary classification problem. The OVO strategy was utilized to extend CSP to multiclass problems. For a problem of N classes, this approach combines each class with every other class in the set, forming $N\times \left(N-1\right)/2$ pairs (three pairs in this study). The CSP was applied to each pair to extract the discriminant features of the given pair for the five frequency bands. Then, the CSP features of all bands were concatenated and input into the classifier. In our study, each trial was classified by three classifiers and labeled three times. Each label was treated as a vote, and the winner class was the one with the maximum number of votes. If each class received the same number of votes, the class with the maximum sum of confidence scores was the final winner class.

### Training and testing phases

**FIGURE 3:**
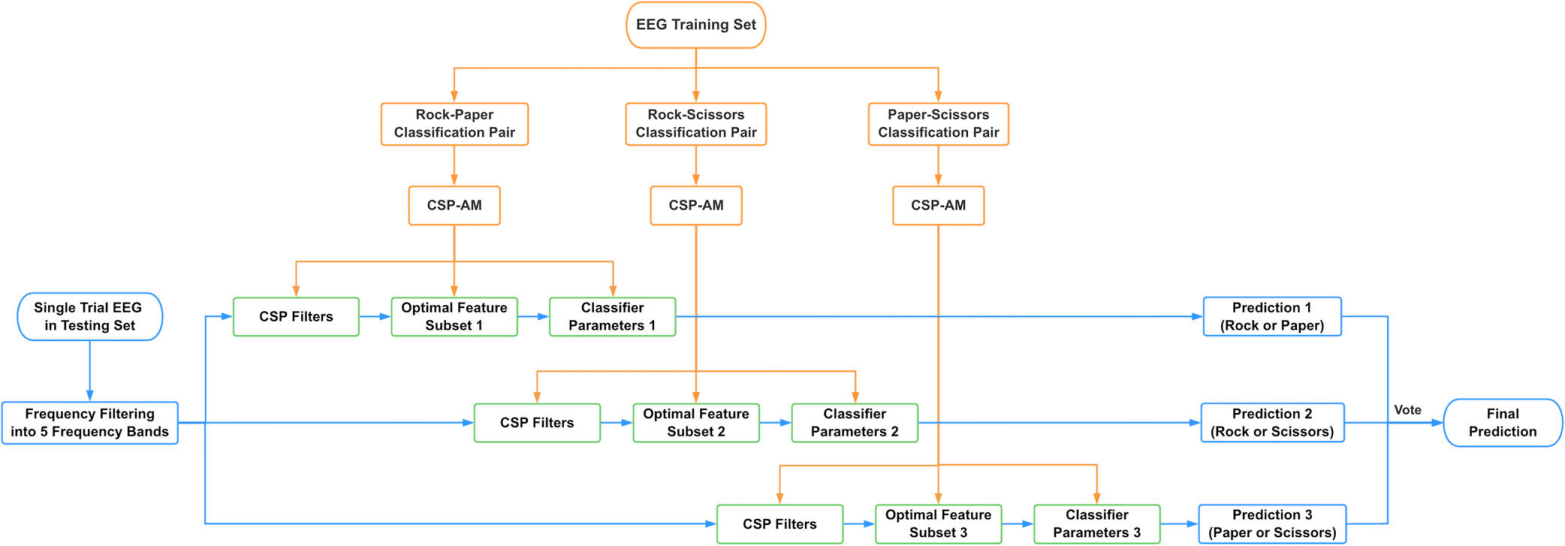
The overall processes of the training and testing phases. The training phase is in orange, and the testing phase is in blue. The green blocks indicate that the output of the training phase is used in the testing phase. CSP, common spatial pattern; CSP‐AM, common spatial pattern‐attractor metagene; EEG, electroencephalogram.

Owing to the large individual differences in EEG data, the training and testing processes were conducted for each participant. Let us denote the number of trials retained after preprocessing for a single participant as N. To simulate an online prediction, the N trials were divided into training and testing sets based on the order of presentation. The first 66.7% of trials served as the training set, and the remaining 33.3% of trials served as the testing set. The overall processes of the training and testing phases are illustrated in Figure 3.

#### Training phase

**FIGURE 4:**
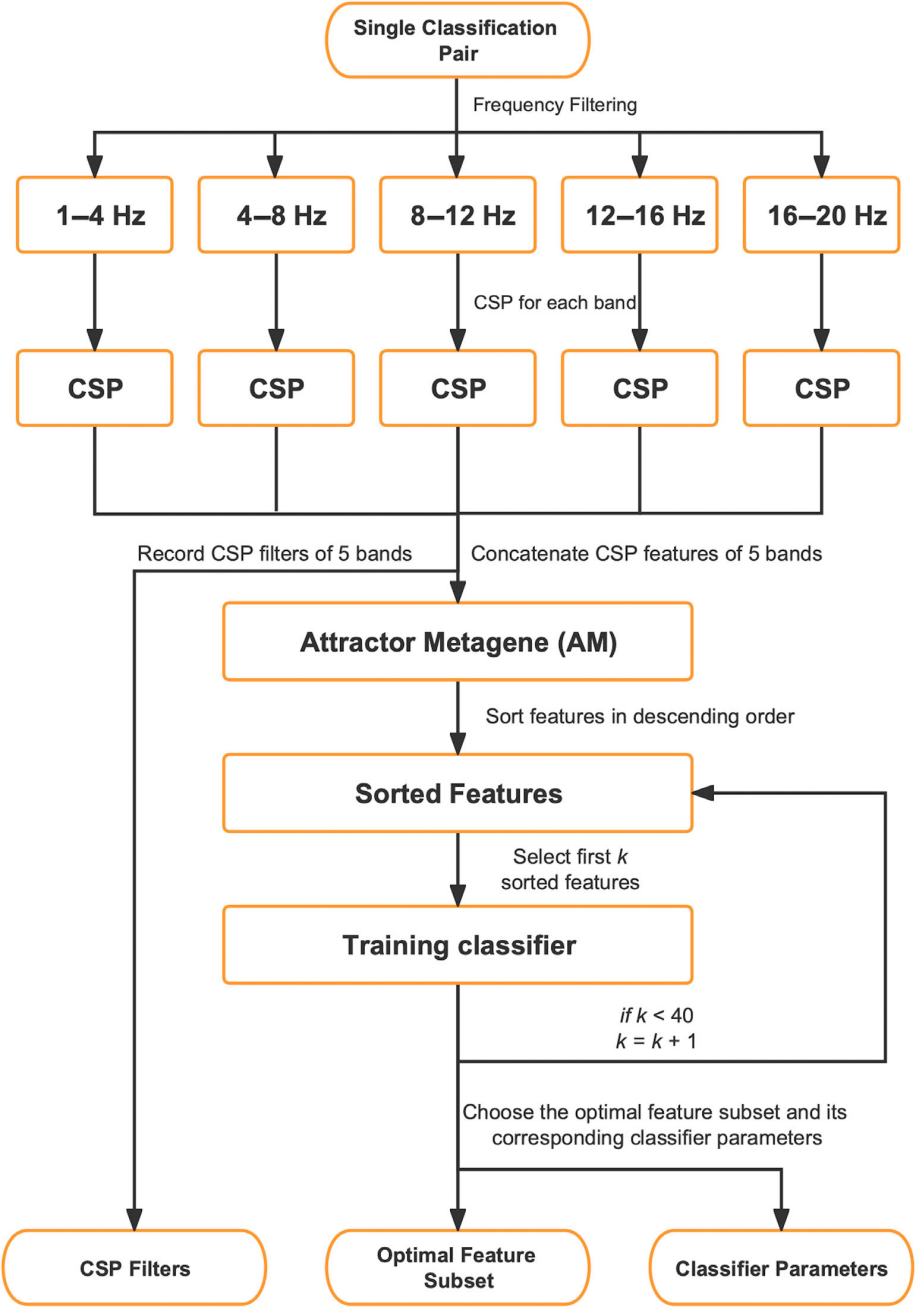
The detailed process of common spatial pattern‐attractor metagene (CSP‐AM) for a single classification pair.

The training phase is illustrated in Figure 3 in orange. In the OVO strategy, the training set was divided into three classification pairs based on pairwise combinations of three different classifications ([rock, paper], [rock, scissors], and [paper, scissors]). The detailed process of CSP‐AM for a single pair is illustrated in Figure 4. Each classification pair was trained separately through the following steps.

1. The CSP was applied locally in each frequency band to extract CSP features.
2. Four pairs of spatial filters were selected from each band. The resulting CSP‐selected features of all five bands were concatenated, forming (5 bands × 4 spatial pairs) 40 features.
3. The 40 CSP features were ranked according to their weight calculated by the AM algorithm.
4. The first k features of the ranked list were selected, where k varies from 1to40.
5. For each pair, the feature subset that provided the best fit, together with its classifier parameters, was maintained.

Hence, the output of the training phase was the CSP filters of five frequency bands, the optimal feature subset that provides the best fit during the 40 iterations and the corresponding classifier parameters. The output was participant‐ and pair‐dependent.

#### Testing phase

The goal of the testing phase is to predict the participant's response in a single trial. The process of the testing phase is illustrated in Figure 3 in blue and proceeded as follows.

1. The corresponding CSP filter was applied to all five frequency bands to extract CSP features, and the features of each band were concatenated to form a 40‐dimensional CSP feature.
2. For each of the three pairs, the features were selected based on the optimal feature subset obtained from the training phase.
3. The selected features were fed into the classifiers with the corresponding parameters.
4. By using the OVO voting principle mentioned above, the final prediction was obtained from the prediction results of the three classifiers.

### Statistical analysis

For comparison, several types of common classifiers, including support vector machine (SVM), decision tree (DT), k‐nearest neighbor (KNN), linear discriminant analysis (LDA), logistic regression (LR), multilayer perceptron (MLP) and random forest (RF), were applied.

Accuracy was calculated for each participant's EEG data in the testing set to evaluate the model's performance. However, we cannot conclude that the accuracy was significantly better than chance merely based on an accuracy value that is higher than chance level (Combrisson & Jerbi, 2015). A nonparametric permutation test was applied to check whether the model had the actual capability to predict decision responses or whether the model exceeded chance level by chance. We randomly permuted the observations across classes and calculated the classification accuracy 10,000 times. The distribution of classification accuracies on random observations can be used to determine significance boundaries for a given rate of tolerated false positives. For instance, if the original classification accuracy is higher than the 95th percentile of the distribution established by randomly permuting the data, then we can conclude that the original classification is significant with p<.05.

We used repeated measures analysis of variance (ANOVA) to compare the accuracies of different classifiers. The statistical significance of accuracy differences among different classifiers was evaluated using the Dunn–Bonferroni post hoc test. The accuracy differences among the three choices within a single type of classifier were also compared using the same method.

To analyze the features extracted from the frequency band that was most frequently selected, we counted the number of times that features in different frequency bands were selected into the optimal feature subset. For each participant, the number of times that features from a single frequency band were selected ranged from 0 to 24 (8 features in a frequency band ×3 classification pairs). We performed Friedman's test and the Conover and Dunn–Bonferroni post hoc tests to reveal possible differences.

## RESULTS

### Performance of classifiers

**FIGURE 5:**
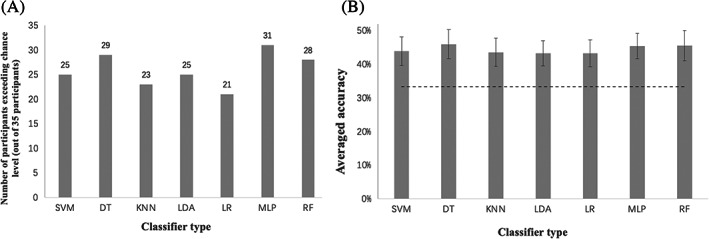
(A) Number of participants significantly exceeding chance level (p<.05) for different types of classifiers. (B) Averaged accuracies of the testing set across 35 participants for different types of classifiers. The dotted line shows the chance level (33.3%). DT, decision tree; KNN, k‐nearest neighbor; LDA, linear discriminant analysis; LR, logistic regression; MLP, multilayer perceptron; RF, random forest; SVM, support vector machine.

After trials with abnormal reaction times had been removed, data from 35 participants were analyzed. For each participant, 276.42 ± 38.94 trials remained, and the latter 33.3% of trials were used as the testing set. The performance of different types of classifiers is shown in Figure 5, and the prediction accuracies on the testing set are shown in Table 1. All the classifiers exceeded chance level (33.33%) by ~10%. The DT classifier achieved the best average accuracy of 45.98%, and the MLP classifier had the greatest number of participants (31 out of 35) that significantly exceeded chance level by the permutation test. All the participants had at least one type of classifier that performed significantly better than chance level, and among 15 participants, all seven adopted classifiers performed significantly better than chance level.

**TABLE 1:** Prediction accuracies of the testing set for each participant and classifier.

Participant number	Accuracy of different classifiers in the testing set (%)						
	SVM	DT	KNN	LDA	LR	MLP	RF
01	38.30	38.30	39.36	44.68**	37.23	43.62*	39.36
02	38.89	48.89**	44.44*	46.67**	40.00	45.56**	50.00**
03	43.12*	42.20*	44.95**	49.54**	44.95**	48.62**	47.71**
04	51.22**	53.66**	48.78**	42.68*	45.12*	45.12*	51.22**
05	43.02*	47.67**	46.51**	45.35**	44.19*	51.16**	48.84**
06	45.63**	42.72*	45.63**	45.63**	42.72*	45.63**	45.63**
07	42.45*	47.17**	48.11**	44.34**	45.28**	42.45*	41.51*
08	55.56**	52.38**	41.27	50.79**	50.79**	55.56**	57.14**
09	39.22	45.10**	39.22	41.18*	40.20	43.14*	40.20
10	37.36	45.05**	41.76*	43.96*	43.96*	38.46	41.76*
11	46.36**	47.27**	46.36**	47.27**	46.36**	48.18**	42.73*
12	47.67**	47.67**	44.19*	48.84**	51.16**	47.67**	44.19*
13	46.15**	47.12**	45.19**	40.38	40.38	41.35*	43.27*
14	45.19**	44.23*	43.27*	43.27*	44.23*	47.12**	43.27*
15	44.14**	44.14**	37.84	36.94	38.74	46.85**	44.14**
16	39.05	43.81*	42.86*	41.90*	42.86*	39.05	47.62**
17	41.94*	50.54**	44.09*	44.09*	44.09*	43.01*	50.54**
18	41.90*	47.62**	44.76**	41.90*	44.76**	46.67**	50.48**
19	41.75*	38.83	42.72*	36.89	39.81	42.72*	42.72*
20	48.96**	53.13**	53.13**	50.00**	45.83**	53.13**	46.88**
21	47.50**	43.75*	45.00*	38.75	46.25**	43.75*	42.50*
22	45.05*	39.56	38.46	40.66	40.66	49.45**	41.76
23	47.73**	48.86**	48.86**	40.91	45.45**	47.73**	54.55**
24	50.52**	53.61**	50.52**	44.33**	45.36**	46.39**	49.48**
25	46.38*	50.72**	43.48*	49.28**	49.28**	43.48*	44.93*
26	41.89	41.89	39.19	43.24*	39.19	44.59*	41.89
27	37.35	43.37**	36.14	42.17*	39.76	39.76	38.55
28	44.44**	40.40	44.44**	37.37	39.39	44.44**	46.46**
29	50.00**	50.00**	53.06**	42.86*	50.00**	47.96**	52.04**
30	43.48*	42.39*	41.30	44.57*	41.30	40.22	43.48*
31	41.24*	48.45**	40.21	41.24*	43.30*	45.36**	49.48**
32	39.44	42.25*	38.03	40.85	33.80	47.89**	46.48**
33	38.46	40.38	39.42	35.58	36.54	47.12**	39.42
34	41.79	44.78*	38.81	41.79	41.79	43.28*	40.30
35	43.12*	51.38**	43.12*	43.12*	48.62**	43.12*	43.12*
AVG	43.89	45.98	43.56	43.23	43.24	45.42	45.53
SD	4.25	4.28	4.15	3.74	4.03	3.69	4.48

*Note*: Accuracies significantly better than chance with p<.05 are marked with an asterisk (*), and accuracies significantly better than chance with p<.01 are marked with two asterisks (**).

Abbreviations: DT, decision tree; KNN, k‐nearest neighbor; LDA, linear discriminant analysis; LR, logistic regression; MLP, multilayer perceptron; RF, random forest; SVM, support vector machine.

Specifically, the SVM classifier reached an average accuracy of 43.89 ± 4.25%, with 25 out of 35 participants significantly exceeding chance level (p<.05). The DT classifier reached an average accuracy of 45.98 ± 4.28%, with 29 out of 35 participants significantly exceeding chance level (p<.05). The KNN classifier reached an average accuracy of 43.56 ± 4.15%, with 23 out of 35 participants significantly exceeding chance level (p<.05). The LDA classifier reached an average accuracy of 43.23 ± 3.74%, with 25 out of 35 participants significantly exceeding chance level (p<.05). The LR classifier reached an average accuracy of 43.24 ± 4.03%, with 21 out of 35 participants significantly exceeding chance level (p<.05). The MLP classifier reached an average accuracy of 45.42 ± 3.69%, with 31 out of 35 participants significantly exceeding chance level (p<.05). The RF classifier reached an average accuracy of 45.53 ± 4.48%, with 28 out of 35 participants significantly exceeding chance level (p<.05).

The accuracy data passed the Shapiro–Wilk test and the sphericity test (*p*s > .05). Repeated measures ANOVA indicated a significant difference between the accuracies of different classifiers ($F\left(6,204\right)=5.632,p<.001,\eta^{2}=0.142$). The post hoc tests (with Bonferroni adjustment) indicated that the DT classifier achieved a significantly higher accuracy than the accuracies of the KNN classifier ($p_{\text{bonf}}=.016$), the LDA classifier ($p_{\text{bonf}}=.003$) and the LR classifier ($p_{\text{bonf}}=.003$). The RF classifier achieved a significantly higher accuracy than those of the LDA classifier ($p_{\text{bonf}}=.029$) and LR classifier ($p_{\text{bonf}}=.030$). The MLP classifier achieved a significantly higher accuracy than that of the LDA classifier ($p_{\text{bonf}}=.049$).

Aside from the DT classifier, the other six classifiers showed no significant difference (ps>.05) in classification accuracies between the three choices (rock, paper, and scissors). However, the DT classifier significantly differed in accuracy among the three choices ($F\left(2,68\right)=26.032,p<.001,\eta^{2}=0.434$). A post hoc test (with Bonferroni adjustment) indicated that the classification accuracy of the scissors choice (M=59.4%) was significantly higher than those of the rock choice ($M=38.2\%,p_{\text{bonf}}<.001$) and the paper choice ($M=39.0\%,p_{\text{bonf}}<.001$). The classification accuracy of the rock choice of the DT classifier showed no difference from the classification accuracy of the rock choice of the other six classifiers ($\;p_{\text{bonf}}s>.05$). The classification accuracy of the paper choice of the DT classifier was significantly lower only than that of the MLP classifier (M=51.1%) and showed no difference from the other five classifiers ($\;p_{\text{bonf}}s>.05$). The classification accuracy of the scissors choice of the DT classifier was significantly higher than that of the other six classifiers ($p_{\text{bonf}}s<.001$).

### Comparison of different frequency bands

**FIGURE 6:**
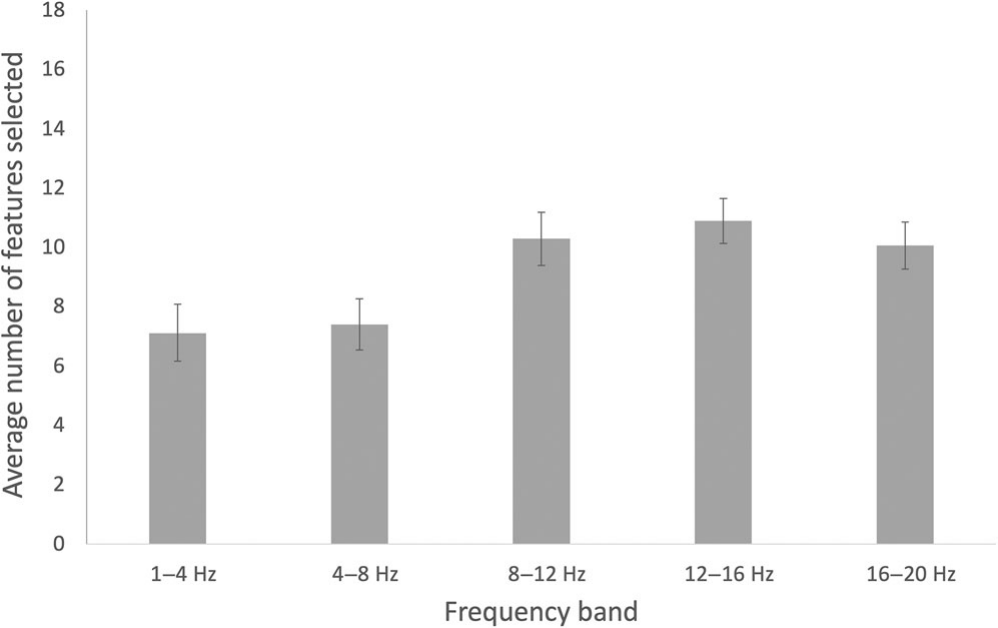
Average number of features selected in different frequency bands across participants. The error bar represents the standard error.

Figure 6 shows the average number of features selected into the optimal feature subset in different frequency bands across participants. Because there were violations of normality under some frequency bands (Shapiro–Wilk test, ps<.05), we used Friedman's test and the Conover and Dunn–Bonferroni post hoc tests to show the significant differences. The Friedman test indicated a significant difference in the number of features selected between different frequency bands ($\chi^{2}\left(4\right)=54.863,p<.001,\text{Kendal}l^{\prime }s\;W=0.392$). The Conover and Dunn–Bonferroni post hoc tests indicated that features extracted from 8–12, 12–16 and 16–20 Hz were significantly more frequently selected than features extracted from 1–4 and 4–8 Hz ($p_{\text{bonf}}s<.01$), while no significant differences were found among 8–12, 12–16 and 16–20 Hz ($p_{\text{bonf}}s>.05$) or between 1–4 and 4–8 Hz ($p_{\text{bonf}}>.05$).

## DISCUSSION

In order to inform online prediction in daily decision‐making scenarios, this study proposed a CSP‐AM algorithm for the prediction of individual choices in a rock–paper–scissors game based on single‐trial EEG signals. CSP features were captured to distinguish different spatial patterns when participants made different decision responses. The AM technique, as an unsupervised hybrid feature‐selection model, was applied to conduct feature ranking efficiently. The findings showed the feasibility of CSP features for multiclass decision‐making prediction and contributed to the development of BCIs for online decision‐making prediction.

The CSP‐AM framework has several advantages regarding the requirements of online prediction. First, the division of the training and testing sets embodies the simulation of the online prediction process. We divided the EEG data of each participant into training and testing sets based on the order of occurrence in the experiment rather than using the widely used cross‐validation technique. The rationale is that cross‐validation would disrupt the temporal order between the training set and the testing set. In an online prediction system, we can only train the model through earlier data to predict individuals' subsequent decision responses. We cannot use future data to predict the past. Based on the aforementioned considerations, we did not use cross‐validation. Second, the algorithm structure was designed to achieve not only high accuracy but also an acceptable running speed and generalizability. Notably, complex ML methods, such as convolutional neural networks, have shown their capability in EEG‐based classification problems offline (Lawhern et al., 2018), but the training times of these methods are too long for conducting online prediction tasks. In addition to classifiers, both the feature extraction and the selection processes are time‐efficient, which makes our approach possible for application in online conditions. Third, the steps of channel and time window selection were consistent among participants, guaranteeing better generalizability. CSPs have been widely used in motor imagery BCI tasks (Nguyen et al., 2018; Yin et al., 2021). These CSP‐based classification methods usually include complex selection procedures of channels (Yin et al., 2021) and time windows (Selim et al., 2018). These individualized steps select different channels and windows for different individuals to reach higher offline classification accuracy but also require more time for the training process and have increased individual differences, which limits their application in online prediction situations.

Another important part of the algorithm is the selection of channels, time windows and features. We used EEG data from all the channels and the same time window (1500 ms before response) for every participant for the reason mentioned above. Based on the interview after the experiment, during the 5‐s “reflection” stage, most participants were referring to the strategy that computers might use and thinking about all three possible choices according to the feedback of the previous trial. They did not make a clear choice until they reached the “countdown” stage. Therefore, we set the time window to 1500 ms before the response. In addition, we removed trials with reaction times less than 100 ms and greater than 1500 ms (i.e., the time between the display of the phrase “please press a button” on the screen and the pressing action). In these trials, participants were considered to be “not thinking at all” or “distracted from the task”. For feature selection, the AM algorithm was adopted for two main reasons. First, the resistance of AM to overfitting has been shown in previous BCI studies (Selim et al., 2018). As mentioned above, the training and testing processes were conducted for each participant, resulting in small datasets. However, overfitting problems are more likely to occur in small datasets. Therefore, AM was utilized to prevent overfitting. Second, the AM algorithm is an efficient feature‐selection method because it provides a ranked list of features. The AM algorithm decreases the time complexity from $O\left(2^{n}\right)$ to $O\left(n\right)$ compared with that of feature‐selection methods that iterate for every feature subset.

For each type of classifier, a great percentile of participants (with a minimum of 21 out of 35 and a maximum of 31 out of 35) achieved classification accuracy significantly better than chance. The results show the classification ability of CSP features in addition to motor imagery problems. As a high‐level cognitive process, decision‐making involves multiple brain regions and the rich associations among them. Spatial filters in the CSP method can distinguish different spatial patterns when people are making different decisions. The DT, MLP and RF classifiers achieved overall higher accuracies than the other classifiers by ~2%. This might be attributable to the advantage of these three algorithms in handling nonlinear data (Breiman, 2001; Cybenko, 1989; Rokach & Maimon, 2013) such as EEG signals. Interestingly, the DT classifier showed a significantly better predictive ability for the scissors choice than for the rock and paper choices, while the other six classifiers showed no significant difference between the rock, paper and scissors choices. We believe that this was due to the randomness of the DT classifier rather than to a special pattern for scissors choices, because the RF classifier (made up of multiple decision trees) showed no difference among the three choices.

However, although the accuracy values for a large percentage of participants were significantly better than chance level, the averaged classification accuracy still needs to be improved for use in real online applications. There may be several factors that limit the improvement in accuracy. First, the continuity of the decision‐making process could lead to a low “information density”. In previous studies using motor imagery or emotion recognition tasks, participants were instructed to keep picturing an image or holding an emotion for a certain period. This relatively clear time window helps increase “information density” and contributes to a higher classification accuracy. In this study, however, participants were not instructed to repeat their choice in their mind after they made it. Although we artificially divided the decision‐making process into several stages, the real cognitive process may not precisely match this division. Thus, it is difficult to fix the time window in which the EEG signals can accurately correspond to a specific cognitive operation. Moreover, the neural activity in the multichoice decision‐making process is more complex than that in the binary‐choice process (Busemeyer et al., 2019; Ditterich, 2010), which brings complexity to the algorithm design and difficulty in achieving higher prediction accuracy. Therefore, we attempted to use the OVO strategy to apply CSP features and the AM algorithm in multichoice decision‐making classification. The results demonstrated that the CSP‐AM algorithm worked well, and an acceptable accuracy significantly exceeding chance level was achieved.

The features extracted from the alpha band (8–12 Hz) and lower beta band (12–20 Hz) were significantly more frequently selected than features extracted from the delta band (1–4 Hz) and theta band (4–8 Hz). We speculate that this is because the cognitive activities involved in the EEG signals from these two frequency bands are more relevant to the rock–paper–scissors task. Previous studies have demonstrated that alpha‐band activity relates to selective attention (Foxe & Snyder, 2011) and controlled access to the knowledge system (Klimesch, 2012). During the rock–paper–scissors decision in our experiment, participants needed to focus on inferring their opponent's strategy based on the results of the last few games and to conceive counterstrategies from their knowledge system. For the lower beta band, previous studies have demonstrated that interactions in the beta band predominate in tasks that strongly involve endogenous top‐down processes (Engel & Fries, 2010). Although the participants were asked to make a choice after a cue, the choice itself was made according to the participants' strategy rather than according to the cue. The decision‐making process was actually a top‐down process determined by endogenous factors, and stimulus features did not deliver any task‐relevant information. Furthermore, beta‐band EEG data have been found to be related to evidence accumulation during decision making (Wyart et al., 2012). During the rock–paper–scissors game in this study, participants needed to continuously accumulate evidence from their opponent's past decisions, infer their opponent's strategies, and thus defeat them.

Some suggestions for future studies are as follows. First, future studies could increase “information density” by adopting other multichoice decision tasks that need specific information offered by the experimenter for participants to make a decision. In such tasks, researchers could clearly identify the time window in which participants were actually thinking about the choices, which is beneficial for increasing classification accuracy. Second, here the participants played a rock–paper–scissors game with a computer player via a screen, which is not a realistic scenario reflecting daily life. The inclusion of a robotic arm would have higher ecological validity and would immerse the participants more deeply in the task.

## CONCLUSION

This study proposed a CSP‐AM algorithm for multichoice decision prediction and verified its performance in predicting a participant's choice in a rock–paper–scissors game based on single‐trial EEG, presenting promising prospects for online prediction in daily decision‐making scenarios.

## CONFLICT OF INTEREST STATEMENT

The authors have no relevant financial or nonfinancial interests to disclose.

## ETHICS STATEMENT

This present study received ethical approval from the Research Ethics Committee of Zhejiang University.
